# Supplementary material for: Determinants of the number of days people in the general population spent at home during end-of-life: Results from a population-based cohort analysis
Source: PLoS One. 2021 Jul 15;16(7):e0253483. doi: 10.1371/journal.pone.0253483 (PMC8282074; doi:10.1371/journal.pone.0253483)
Supplement: S1 File — (PDF) [file pone.0253483.s001.pdf]

Determinants of the Number of Days Spent at Home During End-of-Life Trajectory among General Population: Results from a Population-Based Cohort Analysis

Supplementary File 1

Contents

Supplementary Methods 2

Healthcare registries for retrieving clinical and resource use information 2

Local healthcare programs........ 2

Annual income categories........ 2

Supplementary Figures 3

Figure S1. Flow-chart of individuals included in the analysis. 3

Figure S2. Case distribution for the primary variable (number of days spent at home within the last 180 days of life)........ 4

Supplementary Tables 5

Table S1. Time spent at healthcare and social care facilities within the last six months of life (Women). 5

Table S2. Time spent at healthcare and social care facilities within the last six months of life (Men)........ 9

Table S3. Adjusted models of percentage of days spent at home within the last six months of life for the entire population 13

Table S4. Unadjusted model of percentage of days spent at home. 15

Supplementary Methods

Healthcare registries for retrieving clinical and resource use information

The healthcare registry of the Catalan Ministry of Health, Minimum Basic Dataset (CMBDs, Conjunt Mínim de Dades), includes the following datasets used in this analysis:

- **CMBD-HA.** Contains information from acute care hospitals (i.e., admissions, outpatient major surgery, home hospitalizations, and day hospital).
- **CMBD-SS.** Contains information from inpatient health centers (long- and medium-term stay) and outpatients care teams (PADES).
- **CMBD-AP.** Contains information from primary care teams.
- **CMBD-UR.** Contains information about emergency care.
- **CMBD-SMH and CMBD-SMP.** Contain information from psychiatric hospitals and outpatient mental health centers, respectively.

Local healthcare programs

The PCC and MACA programs are part of the National Strategy for Primary Care and Community Health for achieving patient-centered care. Patients included in the PCC program are those with a chronic disease whose management is considered complex because of either their clinical condition (e.g., multimorbidity, disability, difficult symptom control, and complex management), social environment (e.g., lack of support from family or caretakers, isolated household), or barriers associated with the healthcare organization (e.g., multiple healthcare pathways, lack of hospital-primary care continuum in regular case management). PCC recipients are managed according to a comprehensive and individualized care plan tailored to their health and social care needs. Patients included in the MACA program are those with advance and irreversible chronic conditions that limit their life expectancy between 12 and 24 months; these patients are close surveilled for tailoring healthcare to their rapidly changing needs, including palliative care.

Annual income categories

The socioeconomic status was defined based on the levels of pharmaceutical copayment and divided into four categories: recipient of social benefits (individuals perceiving a minimum integration income, unemployment allowance, unemployment benefit or not qualifying for either of the previous), employed individuals with an annual income of less than € 18,000, between € 18,000 and € 100,000, and more than € 100,000.

Supplementary Figures

Figure S1. Flow-chart of individuals included in the analysis.

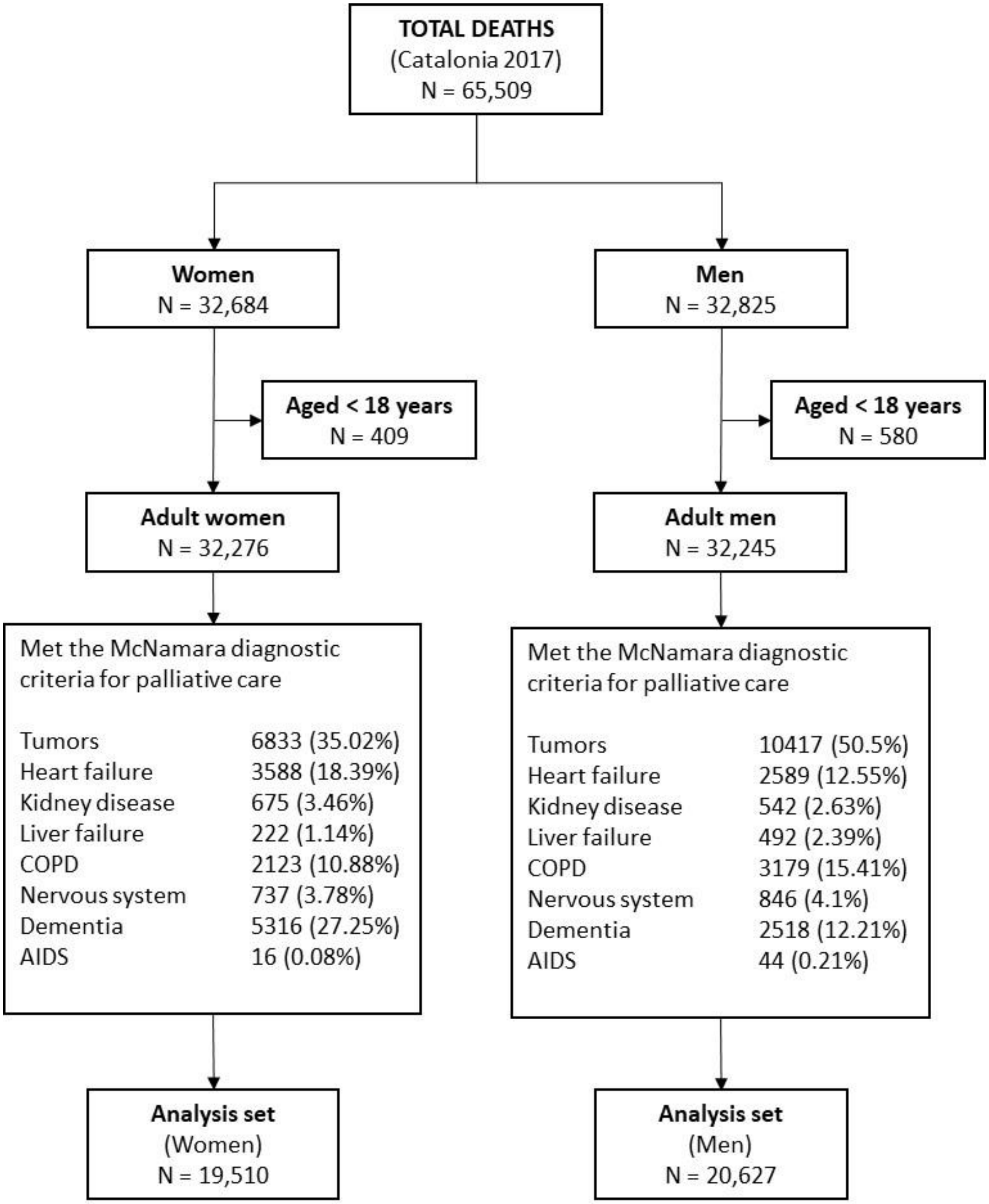

Figure S2. Case distribution for the primary variable (number of days spent at home within the last 180 days of life).
A: women (N=19 510). **B:** men (20 627).

A

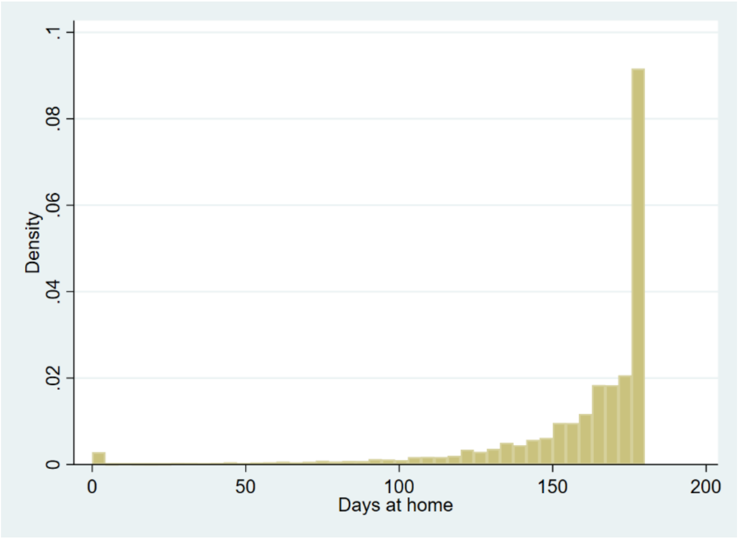

B

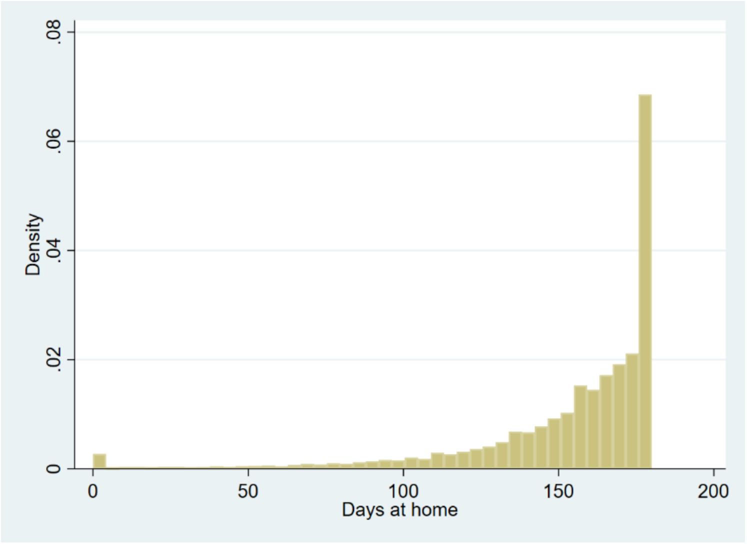

Supplementary Tables

Table S1. Time spent at healthcare and social care facilities within the last six months of life (Women).

	Nursing home		Comprehensive community palliative care service		Out-of-hours urgent primary care services		Emergency medical services		Acute care hospital		Emergency department		Intermediate care facility		Palliative care unit	
	Mean (SD)	Median (IQR)	Mean (SD)	Median (IQR)	Mean (SD)	Median (IQR)	Mean (SD)	Median (IQR)	Mean (SD)	Median (IQR)	Mean (SD)	Median (IQR)	Mean (SD)	Median (IQR)	Mean (SD)	Median (IQR)
**Total**	43.05 (71.79)	0.00 (0.00 - 88.00)	8.22 (28.50)	0.00 (0.00 - 0.00)	0.03 (0.17)	0.00 (0.00 - 0.00)	0.09 (0.29)	0.00 (0.00 - 0.00)	10.32 (15.31)	4.00 (0.00 - 15.00)	0.42 (0.90)	0.00 (0.00 - 1.00)	8.50 (26.54)	0.00 (0.00 - 0.00)	2.60 (9.71)	0.00 (0.00 - 0.00)
**Age (years)**																
<55	2.17 (17.89)	0 (0 - 0)	12.53 (33.17)	0 (0 - 0)	0.01 (0.11)	0 (0 - 0)	0.06 (0.24)	0 (0 - 0)	21.27 (22.80)	15.00 (2.00 - 31.00)	0.57 (1.07)	0 (0 - 1.00)	5.00 (23.72)	0 (0 - 0)	5.17 (14.76)	0 (0 - 0)
55-64	2.06 (16.76)	0 (0 - 0)	12.88 (33.32)	0 (0 - 0)	0.01 (0.12)	0 (0 - 0)	0.06 (0.24)	0 (0 - 0)	18.41 (19.70)	13.00 (3.00 - 26.00)	0.50 (0.94)	0 (0 - 1.00)	4.79 (21.67)	0 (0 - 0)	5.55 (14.53)	0 (0 - 2.00)
65-69	8.54 (35.05)	0 (0 - 0)	13.70 (35.32)	0 (0 - 1.00)	0.01 (0.11)	0 (0 - 0)	0.07 (0.25)	0 (0 - 0)	19.46 (23.10)	13.00 (2.00 - 28.00)	0.47 (0.97)	0 (0 - 1.00)	6.50 (24.65)	0 (0 - 0)	5.11 (14.17)	0 (0 - 1.00)
70-74	13.78 (44.06)	0 (0 - 0)	14.60 (38.14)	0 (0 - 1.00)	0.02 (0.13)	0 (0 - 0)	0.07 (0.25)	0 (0 - 0)	16.59 (18.50)	11.00 (1.00 - 25.00)	0.57 (1.11)	0 (0 - 1.00)	8.53 (26.20)	0 (0 - 0)	4.65 (13.10)	0 (0 - 0)
75-79	24.39 (57.04)	0 (0 - 0)	9.80 (30.79)	0 (0 - 0)	0.02 (0.13)	0 (0 - 0)	0.08 (0.27)	0 (0 - 0)	15.06 (19.18)	9.00 (1.00 - 21.00)	0.48 (0.91)	0 (0 - 1.00)	10.69 (31.17)	0 (0 - 0)	3.66 (11.14)	0 (0 - 0)
80-84	37.98 (68.24)	0 (0 - 29.00)	10.10 (31.22)	0 (0 - 0)	0.03 (0.16)	0 (0 - 0)	0.09 (0.29)	0 (0 - 0)	11.09 (15.05)	6.00 (0 - 17.00)	0.49 (1.01)	0 (0 - 1.00)	9.38 (27.01)	0 (0 - 0)	3.00 (10.19)	0 (0 - 0)

85-89	51.99 (75.47)	0 (0 - 147.00)	6.71 (25.60)	0 (0 - 0)	0.04 (0.19)	0 (0 - 0)	0.10 (0.30)	0 (0 - 0)	8.27 (11.83)	3.00 (0 - 12.00)	0.42 (0.88)	0 (0 - 1.00)	9.64 (27.26)	0 (0 - 0)	1.98 (8.23)	0 (0 - 0)
90-95	61.06 (79.25)	0 (0 - 164.00)	5.42 (24.24)	0 (0 - 0)	0.04 (0.19)	0 (0 - 0)	0.10 (0.30)	0 (0 - 0)	5.84 (9.04)	2.00 (0 - 9.00)	0.35 (0.79)	0 (0 - 0)	8.63 (26.77)	0 (0 - 0)	1.19 (6.04)	0 (0 - 0)
≥95	74.49 (83.08)	1.00 (0 - 174.00)	3.63 (20.02)	0 (0 - 0)	0.04 (0.19)	0 (0 - 0)	0.10 (0.30)	0 (0 - 0)	3.88 (7.07)	0 (0 - 5.00)	0.26 (0.67)	0 (0 - 0)	7.38 (24.50)	0 (0 - 0)	0.83 (4.35)	0 (0 - 0)
**Citizenship**																
Spanish	43.83 (72.22)	0 (0 - 99.00)	8.16 (28.42)	0 (0 - 0)	0.03 (0.17)	0 (0 - 0)	0.09 (0.29)	0 (0 - 0)	10.17 (15.07)	4.00 (0 - 15.00)	0.42 (0.90)	0 (0 - 1.00)	8.53 (26.61)	0 (0 - 0)	2.56 (9.63)	0 (0 - 0)
Foreign	13.01 (43.54)	0 (0 - 0)	12.33 (34.40)	0 (0 - 0)	0.01 (0.10)	0 (0 - 0)	0.06 (0.24)	0 (0 - 0)	18.14 (23.30)	11.00 (1.00 - 26.00)	0.53 (1.03)	0 (0 - 1.00)	6.85 (22.04)	0 (0 - 0)	4.78 (13.18)	0 (0 - 0)
Not available	7.99 (31.25)	0 (0 - 0)	4.59 (17.59)	0 (0 - 0)	0 (0)	0 (0 - 0)	0.06 (0.24)	0 (0 - 0)	10.79 (17.14)	2.00 (0 - 15.00)	0.25 (0.63)	0 (0 - 0)	8.37 (28.40)	0 (0 - 0)	2.77 (8.67)	0 (0 - 0)
**Socioeconomic status**																
Recipients of social services[1]	33.28 (65.18)	0 (0 - 0)	10.59 (33.10)	0 (0 - 0)	0.02 (0.14)	0 (0 - 0)	0.09 (0.29)	0 (0 - 0)	13.47 (17.95)	8.00 (0 - 19.00)	0.47 (0.96)	0 (0 - 1.00)	10.97 (30.71)	0 (0 - 0)	4.12 (12.02)	0 (0 - 0)
<18,000€	47.21 (73.99)	0 (0 - 132.00)	7.65 (27.48)	0 (0 - 0)	0.03 (0.17)	0 (0 - 0)	0.09 (0.29)	0 (0 - 0)	10 (14.89)	4.00 (0 - 14.00)	0.43 (0.91)	0 (0 - 1.00)	8.93 (27.07)	0 (0 - 0)	2.52 (9.65)	0 (0 - 0)
18,000€ - 100,000€	26.52 (59.60)	0 (0 - 0)	10.39 (32.00)	0 (0 - 0)	0.02 (0.15)	0 (0 - 0)	0.07 (0.26)	0 (0 - 0)	11.14 (16.47)	4.00 (0 - 16.00)	0.38 (0.83)	0 (0 - 0)	5.73 (22.00)	0 (0 - 0)	2.64 (9.42)	0 (0 - 0)
>100,000€	10.56	0 (0 - 0)	14.69	0 (0 - 5.00)	0.02 (0.15)	0 (0 - 0)	0.09 (0.29)	0 (0 - 0)	6.72 (11.44)	1.00 (0 - 9.00)	0.31 (0.78)	0 (0 - 0)	4.49 (21.23)	0 (0 - 0)	0.62 (3.39)	0 (0 - 0)

	(39.51)		(35.55)													
Not available	14.26 (43.14)	0 (0 - 0)	5.79 (20.12)	0 (0 - 0)	0 (0)	0 (0 - 0)	0.06 (0.25)	0 (0 - 0)	10.78 (17.39)	2.00 (0 - 14.00)	0.30 (0.69)	0 (0 - 0)	7.10 (26.02)	0 (0 - 0)	2.41 (7.98)	0 (0 - 0)
**Cause of death**																
Tumors	11.42 (40.57)	0 (0 - 0)	16.87 (38.41)	0 (0 - 10)	0.02 (0.14)	0 (0 - 0)	0.06 (0.23)	0 (0 - 0)	14.99 (17.71)	10 (1.00 - 22.00)	0.49 (0.96)	0 (0 - 1.00)	5.75 (21.34)	0 (0 - 0)	5.98 (14.68)	0 (0 - 4.00)
Heart failure	37.17 (67.33)	0 (0 - 28.50)	2.98 (17.51)	0 (0 - 0)	0.03 (0.16)	0 (0 - 0)	0.12 (0.33)	0 (0 - 0)	9.89 (14.03)	4.00 (0 - 15.00)	0.45 (0.90)	0 (0 - 1.00)	9.74 (26.69)	0 (0 - 2.00)	0.74 (4.13)	0 (0 - 0)
Kidney disease	35.53 (66.28)	0 (0 - 6.00)	4.04 (21.10)	0 (0 - 0)	0.03 (0.17)	0 (0 - 0)	0.08 (0.27)	0 (0 - 0)	11.22 (16.43)	6.00 (0 - 15.00)	0.43 (0.82)	0 (0 - 1.00)	11.96 (31.76)	0 (0 - 3.00)	0.83 (4.07)	0 (0 - 0)
Liver failure	15.42 (46.80)	0 (0 - 0)	4.40 (21.51)	0 (0 - 0)	0.01 (0.12)	0 (0 - 0)	0.06 (0.24)	0 (0 - 0)	23.17 (23.66)	17.00 (4.00 - 33.00)	0.79 (1.31)	0 (0 - 1.00)	13.73 (32.52)	0 (0 - 9.00)	1.05 (4.61)	0 (0 - 0)
COPD	48.65 (73.97)	0 (0 - 135.00)	4.11 (21.26)	0 (0 - 0)	0.04 (0.18)	0 (0 - 0)	0.07 (0.26)	0 (0 - 0)	10.50 (15.64)	4.00 (0 - 15.00)	0.46 (0.97)	0 (0 - 1.00)	10.32 (29.17)	0 (0 - 0)	0.93 (5.06)	0 (0 - 0)
Nervous system	52.62 (76.94)	0 (0 - 155.00)	9.33 (33.74)	0 (0 - 0)	0.03 (0.18)	0 (0 - 0)	0.14 (0.34)	0 (0 - 0)	7.56 (14.26)	2.00 (0 - 9.00)	0.34 (0.82)	0 (0 - 0)	10.20 (33.40)	0 (0 - 0)	0.93 (5.17)	0 (0 - 0)
Dementia	86.33 (82.68)	87.50 (0 - 177.00)	2.79 (17.54)	0 (0 - 0)	0.04 (0.20)	0 (0 - 0)	0.11 (0.32)	0 (0 - 0)	4.24 (8.11)	0 (0 - 5.00)	0.30 (0.76)	0 (0 - 0)	9.56 (28.82)	0 (0 - 0)	0.72 (4.27)	0 (0 - 0)
AIDS	6.56 (25.98)	0 (0 - 0)	7.63 (30.50)	0 (0 - 0)	0.06 (0.25)	0 (0 - 0)	0 (0)	0 (0 - 0)	21.44 (24.01)	10 (2.00 - 39.00)	0.44 (0.96)	0 (0 - 0)	18.94 (47.69)	0 (0 - 1.50)	0.19 (0.75)	0 (0 - 0)
**Live wills available**																

Yes	6.69 (30.97)	0 (0 - 0)	17.27 (37.23)	0 (0 - 10)	0.01 (0.11)	0 (0 - 0)	0.08 (0.27)	0 (0 - 0)	15.00 (21.40)	8.00 (1.00 - 22.00)	0.35 (0.70)	0 (0 - 0)	7.01 (20.15)	0 (0 - 0)	5.25 (14.49)	0 (0 - 2.00)
No	43.19 (71.87)	0 (0 - 89.00)	8.18 (28.45)	0 (0 - 0)	0.03 (0.17)	0 (0 - 0)	0.09 (0.29)	0 (0 - 0)	10.30 (15.28)	4.00 (0 - 15.00)	0.42 (0.90)	0 (0 - 1.00)	8.51 (26.56)	0 (0 - 0)	2.59 (9.69)	0 (0 - 0)
**Living alone**																
Yes	59.92 (78.04)	0 (0 - 159.50)	6.09 (24.79)	0 (0 - 0)	0.03 (0.18)	0 (0 - 0)	0.09 (0.29)	0 (0 - 0)	9.19 (12.80)	4.00 (0 - 14.00)	0.43 (0.88)	0 (0 - 1.00)	10.46 (29.04)	0 (0 - 0)	2.25 (9.83)	0 (0 - 0)
No	40.77 (70.60)	0 (0 - 55.00)	8.50 (28.95)	0 (0 - 0)	0.03 (0.17)	0 (0 - 0)	0.09 (0.28)	0 (0 - 0)	10.47 (15.61)	4.00 (0 - 15.00)	0.42 (0.90)	0 (0 - 1.00)	8.24 (26.17)	0 (0 - 0)	2.65 (9.69)	0 (0 - 0)

[1]Includes individuals perceiving a minimum integration income, unemployment allowance, unemployment benefit or not qualifying for either of the previous.

Table S2. Time spent at healthcare and social care facilities within the last six months of life (Men).

	**Nursing home**		**Comprehensive community palliative care service**		**Out-of-hours urgent primary care services**		**Emergency medical services**		**Acute care hospital**		**Emergency department**		**Intermediate care facility**		**Palliative care unit**	
	Mean (SD)	Median (IQR)	Mean (SD)	Median (IQR)	Mean (SD)	Median (IQR)	Mean (SD)	Median (IQR)	Mean (SD)	Median (IQR)	Mean (SD)	Median (IQR)	Mean (SD)	Median (IQR)	Mean (SD)	Median (IQR)
**Total**	18.60 (50.70)	0.00 (0.00 - 0.00)	9.80 (30.76)	0.00 (0.00 - 0.00)	0.02 (0.15)	0.00 (0.00 - 0.00)	0.08 (0.27)	0.00 (0.00 - 0.00)	14.76 (18.90)	9.00 (1.00 - 21.00)	0.52 (1.03)	0.00 (0.00 - 1.00)	8.07 (25.34)	0.00 (0.00 - 0.00)	3.06 (10.42)	0.00 (0.00 - 0.00)
**Age (years)**																
<55	1.25 (12.96)	0 (0 - 0)	7.08 (26.12)	0 (0 - 0)	0.01 (0.09)	0 (0 - 0)	0.07 (0.26)	0 (0 - 0)	21.08 (25.96)	13.00 (2.00 - 30)	0.54 (1.06)	0 (0 - 1.00)	2.98 (17.29)	0 (0 - 0)	3.87 (12.33)	0 (0 - 0)
55-64	3.23 (21.53)	0 (0 - 0)	10.20 (30.14)	0 (0 - 0)	0.02 (0.13)	0 (0 - 0)	0.07 (0.25)	0 (0 - 0)	20.54 (22.29)	15.00 (4.00 - 30)	0.54 (1.05)	0 (0 - 1.00)	5.10 (22.14)	0 (0 - 0)	4.44 (13.40)	0 (0 - 0)
65-69	5.98 (29.45)	0 (0 - 0)	10.39 (31.59)	0 (0 - 0)	0.01 (0.12)	0 (0 - 0)	0.07 (0.25)	0 (0 - 0)	21.52 (23.71)	15.00 (4.00 - 30)	0.54 (1.03)	0 (0 - 1.00)	6.31 (24.28)	0 (0 - 0)	4.48 (12.03)	0 (0 - 1.00)
70-74	8.45 (35.15)	0 (0 - 0)	12.15 (33.43)	0 (0 - 0)	0.02 (0.15)	0 (0 - 0)	0.07 (0.25)	0 (0 - 0)	18.31 (20.67)	12.00 (2.00 - 26.00)	0.62 (1.17)	0 (0 - 1.00)	5.98 (22.28)	0 (0 - 0)	3.78 (10.73)	0 (0 - 0)
75-79	14.11 (44.71)	0 (0 - 0)	10.71 (31.37)	0 (0 - 0)	0.02 (0.13)	0 (0 - 0)	0.07 (0.26)	0 (0 - 0)	17.06 (19.51)	11.00 (2.00 - 25.00)	0.57 (1.07)	0 (0 - 1.00)	8.56 (26.86)	0 (0 - 0)	3.51 (11.17)	0 (0 - 0)
80-84	20 (51.73)	0 (0 - 0)	10.55 (32.33)	0 (0 - 0)	0.02 (0.13)	0 (0 - 0)	0.08 (0.28)	0 (0 - 0)	13.50 (16.60)	8.00 (1.00 - 19.00)	0.53 (1.10)	0 (0 - 1.00)	9.85 (26.57)	0 (0 - 0)	2.68 (8.99)	0 (0 - 0)
85-89	28.16 (60.62)	0 (0 - 0)	9.63 (31.18)	0 (0 - 0)	0.02 (0.15)	0 (0 - 0)	0.09 (0.29)	0 (0 - 0)	10.10 (13.43)	5.00 (0 - 15.00)	0.48 (0.99)	0 (0 - 1.00)	10.41 (27.79)	0 (0 - 3.00)	2.26 (9.45)	0 (0 - 0)

90-95	38.46 (67.86)	0 (0 - 35.00)	7.64 (28.00)	0 (0 - 0)	0.04 (0.19)	0 (0 - 0)	0.09 (0.29)	0 (0 - 0)	7.48 (10.17)	3.00 (0 - 12.00)	0.39 (0.82)	0 (0 - 0)	9.76 (27.64)	0 (0 - 0)	1.76 (8.18)	0 (0 - 0)
≥95	48.91 (74.14)	0 (0 - 133.00)	5.31 (23.23)	0 (0 - 0)	0.04 (0.20)	0 (0 - 0)	0.12 (0.33)	0 (0 - 0)	6.02 (9.30)	1.00 (0 - 9.00)	0.32 (0.76)	0 (0 - 0)	7.36 (20.78)	0 (0 - 0)	0.67 (3.11)	0 (0 - 0)
**Citizenship**																
Spanish	18.97 (51.18)	0 (0 - 0)	9.84 (30.83)	0 (0 - 0)	0.02 (0.15)	0 (0 - 0)	0.08 (0.27)	0 (0 - 0)	14.65 (18.79)	9.00 (1.00 - 21.00)	0.52 (1.03)	0 (0 - 1.00)	8.15 (25.39)	0 (0 - 0)	3.02 (10.35)	0 (0 - 0)
Foreign	7.11 (31.02)	0 (0 - 0)	9.71 (31.09)	0 (0 - 0)	0.01 (0.12)	0 (0 - 0)	0.09 (0.28)	0 (0 - 0)	20.05 (21.55)	15.00 (3.00 - 30)	0.61 (1.13)	0 (0 - 1.00)	5.17 (22.66)	0 (0 - 0)	4.66 (13.30)	0 (0 - 1.00)
Not available	3.52 (17.44)	0 (0 - 0)	3.95 (15.73)	0 (0 - 0)	0 (0)	0 (0 - 0)	0.03 (0.18)	0 (0 - 0)	14.37 (21.81)	8.00 (1.00 - 21.00)	0.26 (0.66)	0 (0 - 0)	5.06 (24.36)	0 (0 - 0)	2.76 (9.50)	0 (0 - 0)
**Socioeconomic status**																
Recipients of social services[1]	19.18 (51.31)	0 (0 - 0)	6.56 (25.60)	0 (0 - 0)	0.02 (0.13)	0 (0 - 0)	0.07 (0.25)	0 (0 - 0)	19.23 (23.19)	12.00 (2.00 - 27.50)	0.63 (1.15)	0 (0 - 1.00)	9.31 (29.37)	0 (0 - 0)	3.61 (11.20)	0 (0 - 0)
<18,000 €	20.88 (53.33)	0 (0 - 0)	9.49 (30.38)	0 (0 - 0)	0.02 (0.15)	0 (0 - 0)	0.08 (0.28)	0 (0 - 0)	14.58 (18.50)	9.00 (1.00 - 21.00)	0.52 (1.04)	0 (0 - 1.00)	8.84 (26.47)	0 (0 - 0)	3.16 (10.57)	0 (0 - 0)
18,000€ - 100,000 €	13.14 (43.38)	0 (0 - 0)	11.22 (32.70)	0 (0 - 0)	0.02 (0.15)	0 (0 - 0)	0.07 (0.26)	0 (0 - 0)	14.73 (19.14)	8.00 (1.00 - 22.00)	0.50 (1.01)	0 (0 - 1.00)	6.04 (21.47)	0 (0 - 0)	2.76 (10.01)	0 (0 - 0)
>100,000€	4.34 (23.58)	0 (0 - 0)	9.16 (26.63)	0 (0 - 0)	0.02 (0.13)	0 (0 - 0)	0.06 (0.23)	0 (0 - 0)	11.54 (18.13)	2.00 (0 - 16.00)	0.30 (0.66)	0 (0 - 0)	1.81 (9.40)	0 (0 - 0)	0.67 (4.17)	0 (0 - 0)
Not available	3.28 (16.85)	0 (0 - 0)	4.42 (15.97)	0 (0 - 0)	0 (0)	0 (0 - 0)	0.04 (0.21)	0 (0 - 0)	14.63	8.00 (1.00 - 21.00)	0.31 (0.89)	0 (0 - 0)	4.71 (23.54)	0 (0 - 0)	2.91 (9.42)	0 (0 - 0)

									(22.61)							
**Cause of death**																
Tumors	6.09 (29.61)	0 (0 - 0)	15.05 (36.47)	0 (0 - 6.00)	0.02 (0.14)	0 (0 - 0)	0.06 (0.23)	0 (0 - 0)	17.20 (19.11)	12.00 (3.00 - 25.00)	0.55 (1.08)	0 (0 - 1.00)	5.46 (20.64)	0 (0 - 0)	5.26 (13.55)	0 (0 - 3.00)
Heart failure	16.79 (48.32)	0 (0 - 0)	2.54 (16.10)	0 (0 - 0)	0.02 (0.14)	0 (0 - 0)	0.14 (0.35)	0 (0 - 0)	11.92 (18.40)	4.00 (0 - 17.00)	0.47 (0.93)	0 (0 - 1.00)	7.13 (22.09)	0 (0 - 0)	0.50 (3.59)	0 (0 - 0)
Kidney disease	19.66 (51.18)	0 (0 - 0)	5.40 (24.41)	0 (0 - 0)	0.03 (0.16)	0 (0 - 0)	0.06 (0.24)	0 (0 - 0)	15.12 (20.68)	8.00 (2.00 - 20)	0.56 (1.05)	0 (0 - 1.00)	9.32 (23.56)	0 (0 - 2.00)	0.96 (5.16)	0 (0 - 0)
Liver failure	4.79 (25.47)	0 (0 - 0)	3.50 (19.39)	0 (0 - 0)	0.02 (0.13)	0 (0 - 0)	0.06 (0.24)	0 (0 - 0)	24.26 (24.88)	18.00 (5.00 - 35.00)	0.70 (1.29)	0 (0 - 1.00)	6.47 (23.08)	0 (0 - 0)	0.97 (5.23)	0 (0 - 0)
COPD	23.00 (55.47)	0 (0 - 0)	6.40 (27.19)	0 (0 - 0)	0.02 (0.15)	0 (0 - 0)	0.10 (0.30)	0 (0 - 0)	15.03 (19.20)	8.00 (1.00 - 22.00)	0.53 (1.06)	0 (0 - 1.00)	11.72 (30.08)	0 (0 - 5.00)	0.98 (5.06)	0 (0 - 0)
Nervous system	32.35 (63.84)	0 (0 - 1.00)	8.50 (31.97)	0 (0 - 0)	0.02 (0.15)	0 (0 - 0)	0.11 (0.32)	0 (0 - 0)	10.93 (20.83)	3.00 (0 - 14.00)	0.39 (0.83)	0 (0 - 1.00)	10.05 (30.46)	0 (0 - 0)	0.94 (4.55)	0 (0 - 0)
Dementia	64.75 (78.57)	1.00 (0 - 164.00)	2.59 (15.93)	0 (0 - 0)	0.03 (0.18)	0 (0 - 0)	0.08 (0.28)	0 (0 - 0)	6.33 (9.91)	2.00 (0 - 9.00)	0.41 (0.86)	0 (0 - 0)	14.39 (34.56)	0 (0 - 9.00)	0.79 (4.97)	0 (0 - 0)
AIDS	5.73 (28.99)	0 (0 - 0)	0.91 (6.03)	0 (0 - 0)	0 (0)	0 (0 - 0)	0.02 (0.15)	0 (0 - 0)	30.05 (28.00)	26.00 (4.00 - 42.50)	0.55 (0.76)	0 (0 - 1.00)	18.09 (44.75)	0 (0 - 0.50)	0.64 (4.22)	0 (0 - 0)
**Living wills available**																
Yes	10.63 (37.93)	0 (0 - 0)	16.99 (37.73)	0 (0 - 14.00)	0.03 (0.17)	0 (0 - 0)	0.07 (0.25)	0 (0 - 0)	17.60 (19.89)	13.00 (2.00 - 28.00)	0.41 (0.84)	0 (0 - 1.00)	9.88 (30.67)	0 (0 - 0)	2.65 (8.40)	0 (0 - 0)

No	18.64 (50.76)	0 (0 - 0)	9.76 (30.71)	0 (0 - 0)	0.02 (0.15)	0 (0 - 0)	0.08 (0.27)	0 (0 - 0)	14.75 (18.89)	9.00 (1.00 - 21.00)	0.52 (1.03)	0 (0 - 1.00)	8.06 (25.31)	0 (0 - 0)	3.06 (10.43)	0 (0 - 0)
**Living alone**																
Yes	36.57 (65.59)	0 (0 - 30.50)	7.22 (27.57)	0 (0 - 0)	0.04 (0.19)	0 (0 - 0)	0.10 (0.31)	0 (0 - 0)	11.67 (15.00)	6.00 (0 - 18.00)	0.49 (0.91)	0 (0 - 1.00)	14.41 (33.99)	0 (0 - 9.00)	2.50 (7.79)	0 (0 - 0)
No	17.60 (49.56)	0 (0 - 0)	9.94 (30.92)	0 (0 - 0)	0.02 (0.14)	0 (0 - 0)	0.08 (0.27)	0 (0 - 0)	14.93 (19.08)	9.00 (1.00 - 22.00)	0.52 (1.04)	0 (0 - 1.00)	7.71 (24.72)	0 (0 - 0)	3.09 (10.54)	0 (0 - 0)

[1]Includes individuals perceiving a minimum integration income, unemployment allowance, unemployment benefit or not qualifying for either of the previous.

Table S3. Adjusted models of percentage of days spent at home within the last six months of life for the entire population

	Minimal adjustment (only gender)					Fully adjusted model				
	%	95% CI	OR	95% CI	*P*	%	95% CI	OR	95% CI	*P*
**Gender**										
Male	69.50	(69.05 - 69.96)	1.00			66.87	(66.39 - 67.36)	1.00		
Female	59.32	(58.76 - 59.87)	0.64	(0.62 - 0.66)	0.00	61.96	(61.44 - 62.48)	0.80	(0.77 - 0.82)	0.00
**Age (years)**										
<55	76.81	(75.67 - 77.96)	1.00			73.01	(71.72 - 74.30)	1.00		
55-64	74.56	(73.65 - 75.47)	0.88	(0.82 - 0.96)	0.00	70.71	(69.70 - 71.72)	0.89	(0.82 - 0.96)	0.00
65-69	70.70	(69.54 - 71.86)	0.73	(0.67 - 0.79)	0.00	66.70	(65.48 - 67.91)	0.73	(0.67 - 0.80)	0.00
70-74	69.77	(68.69 - 70.85)	0.70	(0.64 - 0.76)	0.00	66.77	(65.66 - 67.88)	0.73	(0.67 - 0.80)	0.00
75-79	66.84	(65.79 - 67.89)	0.61	(0.56 - 0.66)	0.00	65.41	(64.38 - 66.44)	0.69	(0.63 - 0.75)	0.00
80-84	64.21	(63.35 - 65.06)	0.54	(0.50 - 0.58)	0.00	64.40	(63.58 - 65.21)	0.66	(0.61 - 0.71)	0.00
85-89	61.04	(60.20 - 61.88)	0.47	(0.44 - 0.51)	0.00	62.77	(61.96 - 63.59)	0.61	(0.56 - 0.66)	0.00
90-95	58.70	(57.71 - 59.70)	0.43	(0.40 - 0.46)	0.00	61.62	(60.64 - 62.60)	0.58	(0.53 - 0.63)	0.00
≥95	54.89	(53.33 - 56.44)	0.37	(0.33 - 0.40)	0.00	58.01	(56.47 - 59.55)	0.50	(0.45 - 0.55)	0.00
**Citizenship**										
Spanish	64.29	(63.93 - 64.66)	1.00			64.40	(64.05 - 64.75)	1.00		
Foreign	71.24	(69.18 - 73.30)	1.38	(1.25 - 1.53)	0.00	66.03	(63.77 - 68.29)	1.08	(0.97 - 1.20)	0.17
**Socioeconomic status**										
Recipients of social services[1]	63.65	(61.87 - 65.42)	1.00			60.00	(58.18 - 61.82)	1.00		
<18,000€	62.77	(62.34 - 63.19)	0.96	(0.89 - 1.04)	0.35	63.34	(62.95 - 63.74)	1.16	(1.07 - 1.26)	0.00
18,000€ -100,000€	70.29	(69.55 - 71.02)	1.35	(1.24 - 1.48)	0.00	69.01	(68.26 - 69.75)	1.52	(1.39 - 1.66)	0.00
>100,000€	81.85	(78.30 - 85.40)	2.60	(2.02 - 3.34)	0.00	78.97	(75.14 - 82.80)	2.63	(2.04 - 3.40)	0.00
**Cause of death**										
Tumors	70.51	(70.06 - 70.97)	1.00			68.45	(67.93 - 68.97)	1.00		
Heart failure	71.68	(70.80 - 72.57)	1.06	(1.01 - 1.11)	0.02	72.85	(72.00 - 73.70)	1.24	(1.18 - 1.31)	0.00
Kidney disease	68.07	(66.04 - 70.10)	0.89	(0.81 - 0.98)	0.02	70.11	(68.14 - 72.07)	1.08	(0.98 - 1.20)	0.12
Liver failure	73.18	(71.10 - 75.26)	1.14	(1.02 - 1.27)	0.02	70.59	(68.40 - 72.79)	1.11	(0.99 - 1.24)	0.06
COPD	63.42	(62.39 - 64.45)	0.72	(0.69 - 0.76)	0.00	64.94	(63.94 - 65.94)	0.85	(0.81 - 0.90)	0.00

Nervous system	60.06	(58.02 - 62.09)	0.63	(0.57 - 0.68)	0.00	59.34	(57.30 - 61.37)	0.67	(0.61 - 0.73)	0.00
Dementia	46.30	(45.34 - 47.27)	0.36	(0.34 - 0.37)	0.00	48.73	(47.73 - 49.72)	0.43	(0.41 - 0.45)	0.00
AIDS	67.49	(59.61 - 75.37)	0.87	(0.60 - 1.25)	0.44	62.45	(54.08 - 70.83)	0.76	(0.53 - 1.10)	0.15
**Living alone**										
Yes	65.40	(65.03 - 65.77)	1.00			65.08	(64.72 - 65.45)	1.00		
No	55.61	(54.26 - 56.95)	0.66	(0.62 - 0.70)	0.00	57.73	(56.46 - 59.00)	0.72	(0.68 - 0.76)	0.00

Table S4. Unadjusted model of percentage of days spent at home.

	Women					Men				
	%	95% CI	OR	95% CI	*P*	%	95% CI	OR	95% CI	*P*
**Age (years)**										
<55	74.01	(72.14 - 75.88)	1			79.51	(78.11 - 80.91)	1		
55-64	75.42	(73.93 - 76.91)	1.08	(0.95 - 1.22)	0.25	75.48	(74.38 - 76.57)	1.08	(0.95 - 1.22)	0.25
65-69	70.07	(68.01 - 72.13)	0.82	(0.72 - 0.94)	0.01	72.61	(71.28 - 73.94)	0.82	(0.72 - 0.94)	0.01
70-74	67.33	(65.37 - 69.30)	0.72	(0.63 - 0.83)	0.00	72.57	(71.33 - 73.81)	0.72	(0.63 - 0.83)	0.00
75-79	64.35	(62.53 - 66.16)	0.63	(0.56 - 0.72)	0.00	69.66	(68.42 - 70.90)	0.63	(0.56 - 0.72)	0.00
80-84	59.91	(58.54 - 61.28)	0.52	(0.47 - 0.59)	0.00	68.21	(67.14 - 69.28)	0.52	(0.47 - 0.59)	0.00
85-89	56.04	(54.84 - 57.24)	0.45	(0.40 - 0.50)	0.00	66.02	(64.84 - 67.19)	0.45	(0.40 - 0.50)	0.00
90-95	54.10	(52.82 - 55.38)	0.41	(0.37 - 0.46)	0.00	63.54	(61.95 - 65.14)	0.41	(0.37 - 0.46)	0.00
≥95	49.67	(47.81 - 51.52)	0.35	(0.31 - 0.39)	0.00	61.80	(58.95 - 64.65)	0.35	(0.31 - 0.39)	0.00
**Citizenship**										
Spanish	59.00	(58.44 - 59.57)	1			69.31	(68.84 - 69.77)	1		
Foreign	69.05	(65.83 - 7s2.28)	1.55	(1.33 - 1.81)	0.00	73.66	(71.06 - 76.26)	1.24	(1.08 - 1.42)	0.00
**Socioeconomic status**										
Recipients of social services[1]	59.43	(56.92 - 61.95)	1			67.44	(64.92 - 69.95)	1		
<18,000€	57.29	(56.66 - 57.93)	0.92	(0.82 - 1.02)	0.11	68.01	(67.46 - 68.57)	1.03	(0.91 - 1.15)	0.66
18,000€ -100,000€	68.39	(67.09 - 69.69)	1.48	(1.31 - 1.67)	0.00	73.06	(72.22 - 73.90)	1.31	(1.16 - 1.48)	0.00
>100,000€	79.16	(72.89 - 85.43)	2.59	(1.75 - 3.85)	0.00	84.49	(80.56 - 88.43)	2.63	(1.91 - 3.63)	0.00
**Cause of death**										
Tumors	69.13	(68.39 - 69.87)	1			72.40	(71.85 - 72.95)	1		
Heart failure	66.05	(64.78 - 67.32)	0.87	(0.81 - 0.93)	0.00	78.04	(76.84 - 79.25)	1.36	(1.26 - 1.46)	0.00
Kidney disease	64.38	(61.43 - 67.33)	0.81	(0.71 - 0.92)	0.00	71.61	(68.84 - 74.37)	0.96	(0.84 - 1.10)	0.58
Liver failure	67.43	(63.27 - 71.60)	0.92	(0.76 - 1.12)	0.43	77.35	(75.10 - 79.60)	1.3	(1.14 - 1.48)	0.00
COPD	58.29	(56.53 - 60.04)	0.62	(0.58 - 0.68)	0.00	67.89	(66.67 - 69.12)	0.81	(0.76 - 0.86)	0.00
Nervous system	54.91	(51.79 - 58.03)	0.54	(0.48 - 0.62)	0.00	64.83	(62.18 - 67.49)	0.7	(0.62 - 0.79)	0.00
Dementia	42.17	(41.00 - 43.34)	0.33	(0.31 - 0.35)	0.00	50.35	(48.70 - 51.99)	0.39	(0.36 - 0.42)	0.00

AIDS	69.31	(53.75 - 84.86)	1.01	(0.48 - 2.10)	0.98	68.90	(60.15 - 77.66)	0.84	(0.56 - 1.27)	0.42
**Living alone**										
No	60.46	(59.87 - 61.05)	1			70.06	(69.60 - 70.52)	1		
Yes	50.86	(49.18 - 52.53)	0.68	(0.63 - 0.73)	0.00	59.44	(57.19 - 61.70)	0.63	(0.57 - 0.69)	0.00

[1]Includes individuals perceiving a minimum integration income, unemployment allowance, unemployment benefit or not qualifying for either of the previous
